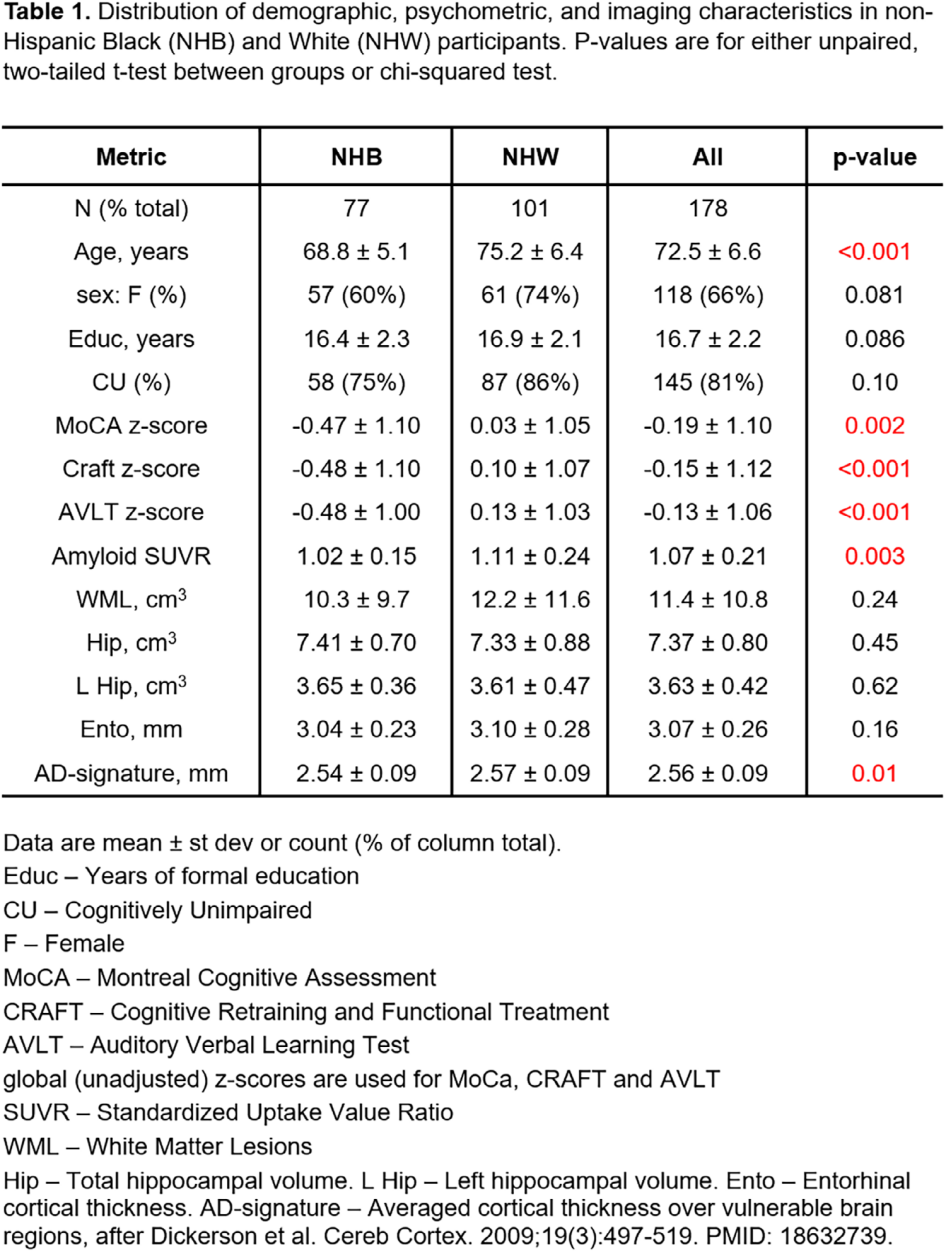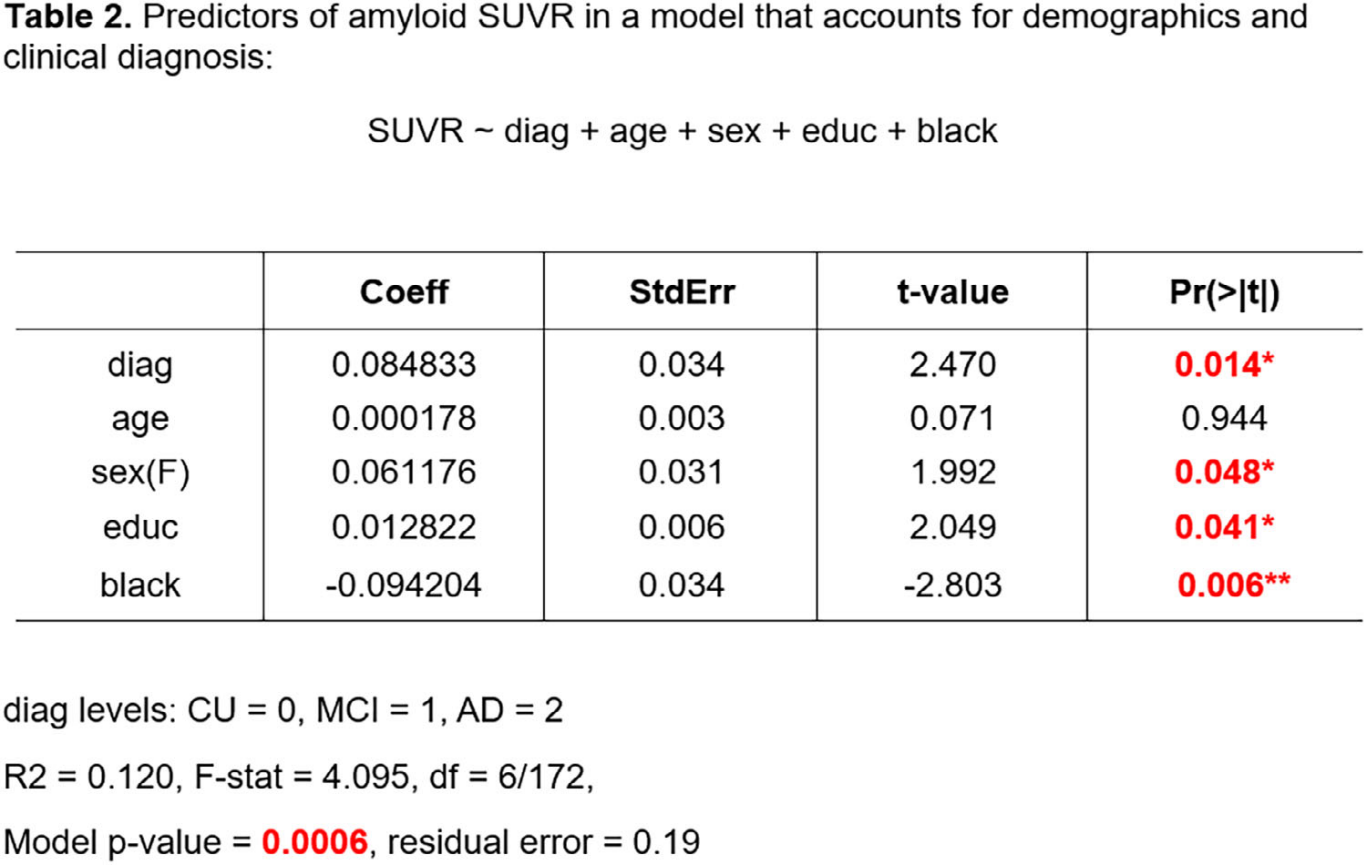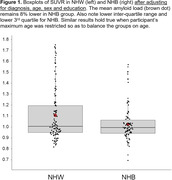# Amyloid burden in non‐Hispanic Black vs White adults: A F18‐florbetaben PET study

**DOI:** 10.1002/alz.091532

**Published:** 2025-01-09

**Authors:** Henry Rusinek, Louisa Bokacheva, Alok Vedvyas, Arjun V. Masurkar, Ricardo S. Osorio, Rebecca A Betensky, Yongzhao Shao, Joshua Chodosh, Karyn Marsh, Thomas Wisniewski

**Affiliations:** ^1^ NYU Alzheimer's Disease Research Center, New York, NY USA; ^2^ Alzheimer's Disease Research Center, New York University Langone Health, New York, NY USA; ^3^ NYU Langone Health, New York, NY, USA, New York, NY USA

## Abstract

**Background:**

Assessment of anti‐amyloid therapies for Alzheimer’s disease (AD) requires understanding racial differences in brain amyloid. We compared amyloid burden in non‐Hispanic Black/African American and White (NHB, NHW) adults in a cohort with over 40% NHB participants and explored factors associated with the inter‐group differences.

**Method:**

We included consecutive non‐Hispanic participants who underwent brain amyloid PET‐MRI exams in 2021‐2023 at NYU Alzheimer's Disease Research Center. PET images were acquired on Siemens 3T PET‐MR system after an injection of 300 MBq of ^18^F‐florbetaben, reconstructed and normalized by the mean activity of the whole cerebellum. Composite SUVR was calculated by averaging frontal, parietal, temporal and cingulate cortical regions (Bullich 2021 PMID:33773598) segmented using FreeSurfer 7.1. White matter lesion (WML) volume was measured on FLAIR images. We used linear regression to test amyloid SUVR for association with demographic, cognitive, and MRI features.

**Result:**

A total of 178 participants were enrolled (age, 72.5 ± 6.6 years old; 66% female), including 77 (43%) NHB and 101 (57%) NHW (Table 1). NHB were significantly younger than NHW, showed significantly lower memory and cognitive performance, yet also had significantly lower mean value and third quartile of amyloid SUVR distribution (Figure 1). Most measures of brain atrophy and white matter lesion load were not significantly different between groups.

Regression analysis showed that race was the strongest predictor of SUVR. This finding remained true in the regression model adjusted for age, sex, education and clinical diagnosis (Table 2).

**Conclusion:**

Consistent with two prior studies (Xiong 2022 PMID:35218143, Deters 2021 PMID: 33568538), we found differences in amyloid SUVR distribution and an unexpected relationship to memory performance in NHB compared to NHW. The finding may stem from several factors, including social/environmental differences, cohort selection biases, cognitive reserve and educational attainment, genetics, and different resilience to amyloid burden. In our cohort, NHB and NHW had comparable volume of WML, therefore cerebrovascular disease was unlikely the primary driver. Since lower amyloid burden in NHB may create bias in inclusion in anti‐amyloid treatment trials, it merits further investigations to be better understood.